# Resistance and Resilience of Species Composition: Thirty Years of Experimental Mismanagement and Subsequent Restoration in a Species Rich Meadow

**DOI:** 10.1002/ece3.70923

**Published:** 2025-01-29

**Authors:** Jan Lepš, Aleš Lisner

**Affiliations:** ^1^ Department of Botany, Faculty of Science University of South Bohemia České Budějovice Czech Republic; ^2^ Biology Center of the Czech Academy of Sciences Institute of Entomology České Budějovice Czech Republic

**Keywords:** abandonment, competition asymmetry, dominant removal, fertilization, global warming, mowing, resilience, resistance

## Abstract

Traditionally managed grasslands are among the most species‐rich communities, which are threatened by land use changes—management intensification or abandonment. The resistance of their species composition to mismanagement and ability to recover after re‐establishment of traditional management is of prime conservational interest. In a manipulative experiment in a wet meadow, we simulated mismanagement by a factorial combination of abandonment of mowing and fertilization. The dominant species *Molinia caerulea* was removed in half of the plots to assess its role in community dynamics. The 21 years' mismanagement period was followed by the re‐establishment of the traditional management. The plots were sampled yearly from 1994 (the baseline data, before the introduction of the experimental treatments), until 2023. Estimates of cover of all vascular plant species provided the species richness and effective number of species. For each year, the chord distances to baseline species composition and to corresponding control plot were calculated. The compositional data were analyzed by constrained ordination methods, and the univariate characteristics by Repeated Measures ANOVA. All the plots, including those with traditional management throughout the whole experiment, underwent directional changes, probably caused by a decrease in groundwater level due to global warming. Both fertilization and abandonment led to a loss of competitively weak, usually low‐statured species, due to increased asymmetric competition for light. The effect of fertilization was faster and stronger than that of abandonment demonstrating weaker resistance to fertilization. The removal of dominant species partially mitigated negative effects only in unmown, non‐fertilized plots. The recovery following mismanagement cessation was faster (signifying higher resilience) in unmown than in fertilized plots, where it was slowed by a legacy of fertilization. In a changing world, two reference plot types are recommended for assessment of resistance and resilience, one original state and one reflecting compositional changes independent of treatments.

## Graphical abstract

In a 30‐years manipulative experiment in traditionally managed species rich oligotrophic meadow, we demonstrated how the community composition and diversity deteriorated under mismanagement (abandonment and fertilization), and how it recovered after re‐introduction of the traditional management. The composition of the community, regardless of the treatment combination, underwent directional changes (shown by partial RDA below), probably caused by a decrease in underground water level due to global warming. The consequences for the compositional resistance and resilience of the community are discussed.

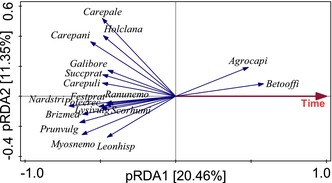

## 1 Introduction

Stability is one of the most studied properties of communities and ecosystems (Hautier et al. 2020). It has long been accepted (Pimm 1984) that ecological stability has different facets. The most commonly studied ones are *constancy* (reciprocal of variability, sometimes called invariability), which characterizes fluctuations “under normal conditions,” resistance, and resilience. Resistance is the ability to tolerate, remaining unchanged, some perturbation period or extreme event (usually defined as significant deviation from normal conditions), while resilience (recovery) is the ability to return to original state after the perturbation (de Bello et al. 2021; Pimm 1984). The time scale at which the responses are studies affects the stability assessments. In experimental studies, the perturbation period is imposed artificially, and its effect compared to controls without this perturbation. This enables separation of the perturbation effect from the effects of uncontrolled variation in external conditions.

Constancy is typically characterized by its reciprocal, that is, variability. The coefficient of variation, commonly used metric of variability, reflects the fact that we expect some “reference state”, around which the community fluctuates. However, many communities undergo directional changes (e.g., long‐term succession, or changes forced by long‐term environmental change, e.g., climate warming). For resistance and resilience, theoretical models expect some type of equilibrium (Arnoldi et al. 2018), but in reality, even the controls without perturbation might undergo temporal trend or random fluctuations, and we need to reflect them in resistance/resilience assessment.

Although it is self‐evident and has been recognized for a long time (Pimm 1984; Koblihová‐Baumová et al. 1990; Lepš 1990), it has only in recent years been sufficiently appreciated that any facet of stability is always related to the state variables characterizing the community (Hillebrand and Kunze 2020; Polazzo and Rico 2021). The most frequently used characteristics are aggregated ones, which characterize community functioning, for example, total biomass, frequently used to characterize productivity. In many cases (typically in conservation programs), the stability of the community's species composition, or of characteristics based on the species composition (functional composition, species and functional diversity) are of interest. The resistance and resilience of total abundance and species composition might be rather different (Lepš 1990, p. 199; Lepš, Osbornová‐Kosinová, and Rejmánek 1982; Lipoma et al. 2016). Recent theories and experimental studies demonstrating the importance of asynchrony for stability of total community abundance (Liu et al. 2018; Valencia et al. 2020) assume that one of the ways to achieve stability in aggregated characteristics is through the compositional instability (stability of yearly productivity is achieved through changes in species proportions). While some approaches attempt to integrate various stability facets into one measure (Urrutia‐Cordero et al. 2022), in many practical cases we are interested in one select facet, or in comparison of stability based on different state variables.

Most studies compare the stability characteristics of different communities (Bazzichetto et al. 2024), but less attention has been paid to the fact that both resistance and resilience are also determined by the type and strength of the perturbation period. A study of differently aged successional stages in old fields has shown that comparison of resistance and resilience depended on both, type of experimental perturbation and the state variables (Lepš 1990), in contrast to the constancy, which was higher in the older fields regardless of studied state variables. We are also interested in the extent to which stability is affected by the (few) dominant species, and to which extent it is a function of community assembly characteristics, typically community diversity (Lisner et al. 2024).

Semi‐natural temperate meadows belong to the most species rich communities (Chytrý et al. 2015), hosting many endangered species, which often lost their natural habitats in highly cultural Central European landscape. They are dependent on traditional management (mowing once or twice a year, no or very limited fertilization), which is no longer economically viable. Their management is thus intensified (including fertilization) or they are abandoned, and then sometimes eutrophicated from surrounding arable fields. For these communities, species composition stabilized under the decades and probably even centuries of the traditional management, and both, the fertilization and abandonment should be considered as perturbation. All these changes lead to a decline in diversity, accompanied by the local extinction of species of conservation interest (Isselstein et al. 2005). From the biodiversity conservation point of view, all these changes can be considered as mismanagement. If the traditional management is reintroduced (usually as a part of conservation measures), the return to an “original” species rich state may be hindered by a legacy of mismanagement (e.g., remaining nutrients after fertilization), and very slow (Isbell et al. 2013; Seabloom et al. 2020; Spiegelberger et al. 2006). It can also be slowed by priority effects, where dominants established during the perturbation period have an advantage in asymmetric competition with returning species (Fukami 2015).

In addition to land use changes, continuous climate change might also change the species composition, irrespective of changes in management (Liu et al. 2018). It has been recently shown that most of the Central European non‐forest vegetation undergoes directional changes (Klinkovská et al. 2025). The management practices might be realistically simulated by experiments, and contrasted with appropriate control; on the contrary, we do not have any control to contrast with the omnipresent global warming. We established a long‐term management experiment in a species rich, traditionally managed meadow. The study mimicked common mismanagement (from a conservation point of view) for a 21 years' perturbation period, which included factorial combination of fertilization and abandonment of mowing. After 21 years, the mismanagement was ceased and replaced by traditional management. To investigate the role of dominant species, we also added a dominant removal treatment.

Our aim is to compare resistance of community diversity and composition to various types of conservation mismanagement and its resilience characterized by 8 years of recovery under “traditional management.” In addition, the 30 years' time series enables us to test for a possible common trend, independent of the experimental treatment, and reflect it in the resistance and resilience assessment.

## 2 Methods

### 2.1 Study Site

**FIGURE A1:**
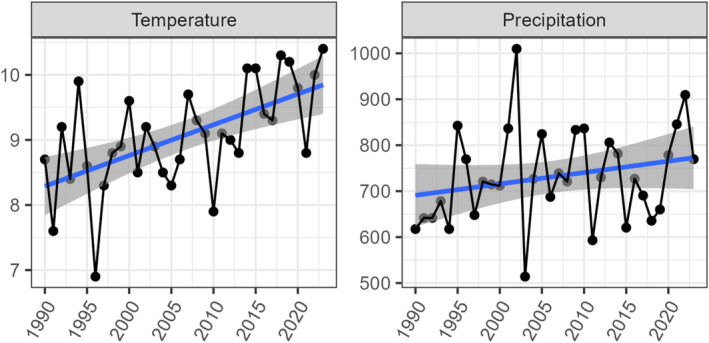
Changes in the mean annual temperature and yearly precipitation (mm.year^−1^) since 1990 (the experiment started 1994). Linear trends are shown, the trend in temperature is significant (*p* < 0.001), the trend in precipitation is not (*p* = 0.165).

The experiment was established in the oligotrophic, species rich meadow in South Bohemia, Czech Republic (48°57′N, 14°36′E, altitude 510 m) in 1994 (Lepš 2014). The average annual temperature has steadily increased by 1.9°C, rising from 7.9°C to 9.8°C between 1979 and 2023. Within the study timeframe (1994–2023), this corresponds to an average increase of approximately 1.4°C and the trend is significant (*p* < 0.001). Mean annual precipitation has also shown an increase of 31 mm, increasing from 719 mm to 750 mm. However, within the study timeframe, the increase was even bigger, corresponding to a 74 mm increase, though, not significant (Figure A1 in Appendix). The minimum and maximum yearly average temperatures recorded were 6.9°C and 10.4°C respectively, and the precipitation ranged between 514 and 1010 mm. These data were extracted from the Climate Change meteoblue model, which utilizes data from ERA5, the fifth generation ECMWF atmospheric reanalysis of the global climate (accessible at: https://www.meteoblue.com/en/climate‐change/ohrazen%c3%ad_czechia_3069123).

The plant community is an oligotrophic moderately wet species‐rich meadow (over 30 species of vascular plants per m^2^) dominated by the tussock grass *Molinia caerulea* . Other abundant species are mostly grasses ( *Festuca rubra* , *F. ovina* , *Holcus lanatus* , *Danthonia decumbens*, and many others) and about 10 species of sedges (*Carex* spp.). Dicots are numerous, but usually not dominant (*Potentilla erecta, Ranunculus acris, Succisa pratensis, Betonica officinalis*), see Table A1 for other common species. Similar meadows in the region have traditionally been extensively mown (Isselstein, Jeangros, and Pavlů 2005). The site was mown regularly until the late 80s, such that at the start of the experiment, the meadow was ~3–4 years without mowing.

### 2.2 Experiment

**FIGURE A2:**
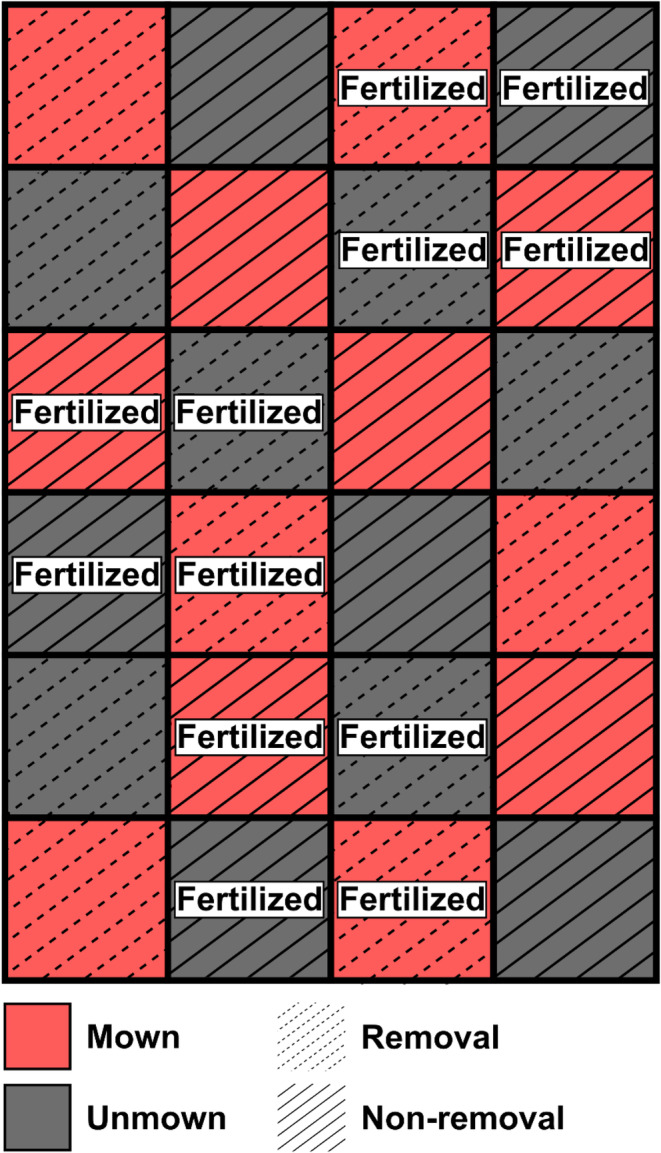
Design of the experiment. The dimensions of each quadrat are 2 × 2 m. Colors and line types correspond to the graph legends in the main text and to most of the legends in the appendix.

In 1994, 24 permanent mown plots were established, each 2 × 2 m, in a lattice consisting of six rows and four columns, adjacent to each other. Fertilization, mowing, and dominant removal were applied as treatments in a factorial design, forming eight treatment combinations, alternating regularly in the lattice (each couple of rows contained all the treatment combinations, so each combination was replicated three times; Figure A2). Experimental mowing was done by sickle during June of each year. Initially, the fertilization treatment consisted of annual applications of 65 g/m^2^ of a commercial NPK fertilizer (12% N as nitrate and ammonium, 19% P as P_2_O_5_, and 19% K as K_2_O, corresponding to 7.8 g of N, 12.4 g of P, and 12.4 g of K per m^2^). The experiment was originally planned as a shorter term one; after 8 years, and after 17 year again, we decided to prevent the unrealistic accumulation of phosphorus in the soil by decreasing the dosage, finally (from 2012 onwards corresponding to 4 g of N, 6.5 g of P, and 5.5 g of K per m^2^). From conservation point of view, it is still the same mismanagement with high soil fertility. *Molinia caerulea* tillers were manually removed with a screwdriver in April 1995, attempting to minimize the soil disturbance, and new individuals of *M. caerulea* were removed annually as needed. Seedlings of woody species were removed in all plots when they appeared (although the encroachment of meadows by woody species is a problem for conservation, the encroachment is too much dependent on the landscape context, mainly vicinity of seed source, and 2 × 2 m quadrat is not a proper spatial scale for studying woody encroachment). A detailed description of the locality and experiment is in former summaries (Lepš 1999, 2014) of the experiment. Since 2016, the traditional management (no fertilization, mowing) was re‐established in all the plots. Using the general resistance/resilience research terminology (Isbell et al. 2015), the 1995–2015 mismanagement is the 21 years' perturbation period of press type (Polazzo and Rico 2021), and 2016–2023 is the post‐perturbation recovery. *Molinia* removal continues to see also the dependence of recovery on the presence of a dominant.

### 2.3 Monitoring

In the center of each plot, a permanent quadrat 1 × 1 m was established and permanently marked (this way there was 0.5 m buffer zone) (non‐sampled) on each side. Plots were sampled (visual estimates of cover of all the species) each year in late May/early June by the same person (JL). The first sampling was done in 1994 before treatments were introduced, which provided an estimate of random variability in species composition in the plots (useful as a yardstick for possible effects of treatments later on), and baseline data for analyses.

### 2.4 Data Analyses

For the analyses, we used complete species composition of vascular plants from each plot (24 plots × 30 years = 720 relevés). We characterized the diversity of each relevé by species richness (number of species per relevé, *S*) and effective number of species (ENS), *N*_2_ in unified Hill's notation (Hill 1973), which reflects both the species number and the evenness of species cover values.

$$\mathrm{ENS}=\frac{1}{\sum_{i=1}^{S}p_{i}^{2}}$$

where *p*_*i*_ is the proportion of *i‐*th species. For the diversity characteristics, we included all species, including the manipulated *Molinia* (omitted for the analyses of composition, see below). This did not make a substantial difference in the number of species, because *Molinia* was present in most plots, including the removal plots (usually as only a few tillers). However, it did affect the ENS, because in the non‐removal plots *Molinia* was often the dominant. By comparing corresponding treatments with and without removal, we ask whether the remaining species after removal formed a similar abundance distribution as the original community. We further used the cover of *Molinia* as an important community characteristic and analyzed its changes.

The data on species composition were log(x + 1) transformed (cover as a percentage) and analyzed by ordination methods. In all the analyses the manipulated dominant, *Molinia caerulea*, was omitted (we were interested in the reaction of the rest of the community, not in the fact that *Molinia* was missing when removed). First, we used CCA (Šmilauer and Lepš 2014) with the interaction of the treatment (i.e., eight categories, factorial combinations of the three binary factors) with year as a categorical variable and then Principal Response Curves (PRC; Van Den Brink and ter Braak 1999), with the standardization by sample norm. In CCA, the explanatory variables have 30 (years of the experiment) × 8 (treatment combinations)—1 = 239 degrees of freedom (for 720 observations). This means that although CCA is a constrained analysis, the constraint is very weak, but the results are focused on the differentiation of the dynamics among individual treatments. The trajectories of individual treatment centroids in the ordination diagram show both the treatment differentiation and the possible common temporal trend (as the year is a categorical variable, the constraint cannot introduce temporal trend by itself). With these settings, the CCA enables to compare in the same ordination diagram the response of community composition to the treatments with the common dynamics independent of the experimental treatments. PRC uses time (categorical) as a covariate, which means that any variability among years (including possible common temporal trend) is partialled out. PRC is focused on comparison of treatments with the reference within each time; mowing, no fertilization, no removal combination (i.e., traditional management) was selected as the reference level. Because the results of CCA suggested a common temporal trend, we further tested this trend directly in plots, which kept their management unchanged during the whole period (i.e., the mown unfertilized plots without removal). Time since the start of the experiment was the only explanatory variable for this analysis and plot identity was the covariate, meaning that the results reflect only the temporal trend. The permutations were within plots, concordant over all the plots. Because the species composition variability in mown unfertilized plots over time was much lower than in the whole data set, the RDA (centering by species, standardization by sample norm) is the appropriate constraint ordination method here (Šmilauer and Lepš 2014). Note that in all the ordination methods, the variability explained by individual ordination axes includes also reduction of the dimensionality and so the values are usually much lower than usual values of *R* ^2^ in univariate analyses (Šmilauer and Lepš 2014).

To directly characterize the displacement of species composition by the imposed treatments, we calculated for each relevé its chord distance to the reference state. Chord distance is the Euclidean distance with species composition standardized by sample norm; 0 means identical species proportions, $\sqrt{2}$ means no common species, see Šmilauer and Lepš (2014). With respect to the possibility of a common trend in the data, we used two different reference states: (1) the baseline observation in 1994 for each quadrat and each year (showing how much the given quadrat changed from the baseline) and (2) the control plot (mowing, no fertilization, no removal) showing how much given quadrat in given year differs from spatially closest control. All the species, excluding the manipulated *Molinia*, were used for the calculation of distances. Cover was log(x + 1) transformed.

Although the detailed analysis of changes in species traits is outside of the scope of this paper, we did choose to calculate the community weighted mean (CWM) for vegetative height, the most responsive trait (Lepš 1999), and used it to illustrate community change in time. For each species we used a fixed value, composed of the average of values from all treatments from Mudrák et al. (2019), sampled in the experiment in 2013; changes in CWM thus correspond to species turnover.

For the univariate responses (cover of *Molinia*, species richness, chord distance, CWM), we used Repeated Measures ANOVA. Based on this model, we calculated the standard error for between and within plot comparisons from the corresponding mean squares as $\mathrm{SE}=\sqrt{\frac{{\mathrm{MS}}_{e}}{2}\left(\frac{1}{n_{A}}+\frac{1}{n_{B}}\right)}$, where *MS*_e_ is the corresponding error mean square and *n*_A_ and *n*_B_ are the numbers of replications in the compared groups (i.e., 3) and graphed it in figures. *MS*_e_ was obtained from the repeated measures ANOVA model—for the comparison of treatments, we used the between plots error MS, whereas for changes in time we used the remainder (within plot) MS.

All the univariate analyses and graphical outputs were performed using R software version 4.3.3 (R Core Team 2024), and ordinations using Canoco5 (Šmilauer and Lepš 2014).

## 3 Results

**FIGURE A3:**
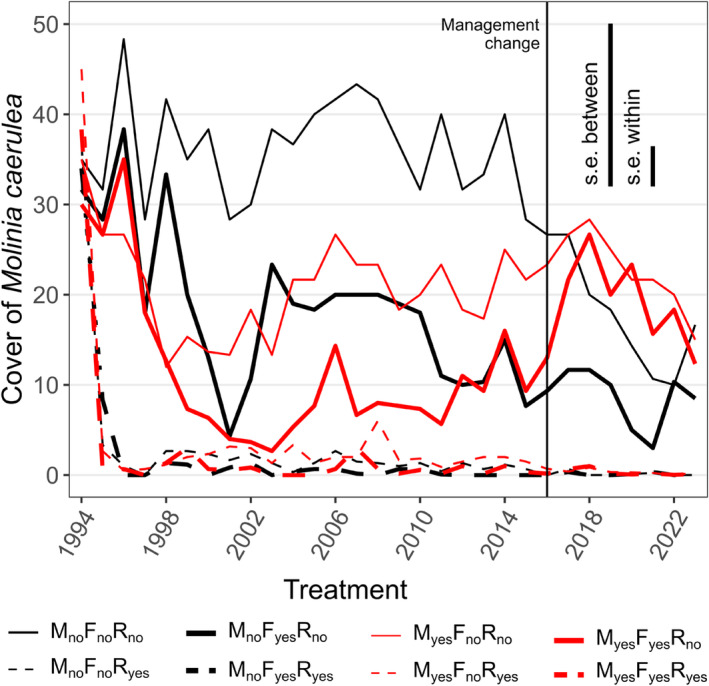
The temporal changes in cover of *Molinia*. Results of corresponding Repeated measures ANOVA are in Table S2. The heavy lines mean fertilization (light = no fertilization), the red lines mean mowing (black = unmown) and the broken lines mean *Molinia* removal. The vertical lines mean standard errors for between and within plot comparisons, derived from the Repeated Measures ANOVA model (Table S3). In the model, only the non‐removal plots are analyzed. The re‐installment of original traditional management in 2016 is shown by thin vertical line.

The removal of *Molinia* successfully reduced its cover to minimal levels. In plots where the species was not removed it responded to the treatments—*Molinia* was suppressed by fertilization, but also by mowing and attained highest cover in unmown unfertilized plots (Figure A3, Table A2). After the re‐introduction of traditional management, however, it sharply decreased. Interestingly, there was also some initial decrease of *Molinia* in traditionally managed (mown, unfertilized plots).

**FIGURE 1:**
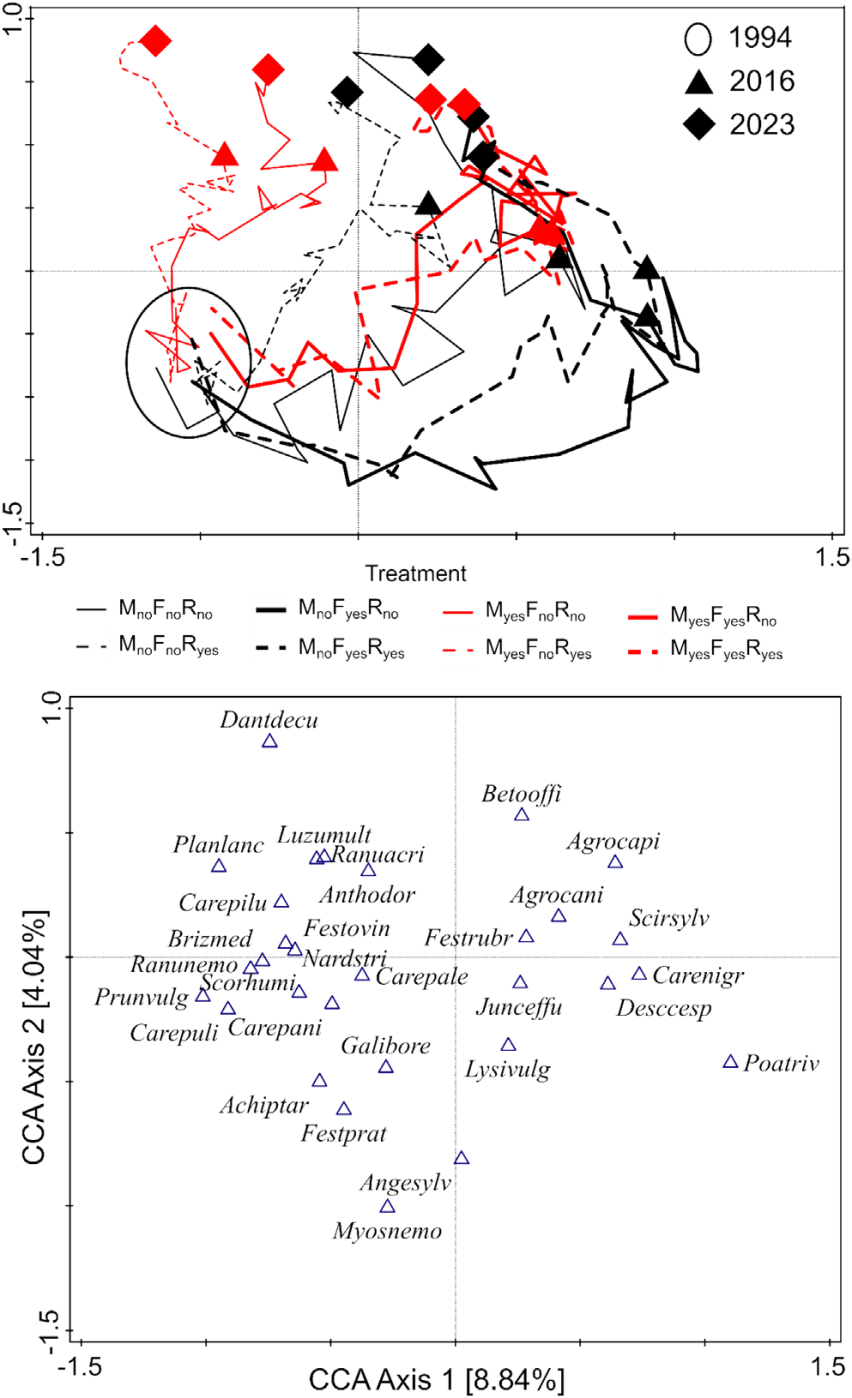
Ordination diagram of CCA with the interaction of treatment × time being the explanatory variable. The upper panel shows the trajectories of centroids of the treatment combinations, and the lower panel the corresponding ordination of species (the best fitted species were selected for the display). The variability explained by individual axes is presented in brackets. The heavy lines mean fertilization (light = no fertilization), the red lines mean mowing (black = unmown) and the broken lines mean *Molinia* removal. 2016 is the year of re‐establishment of traditional management. Full species names for the acronyms are in Table A1.

**FIGURE 2:**
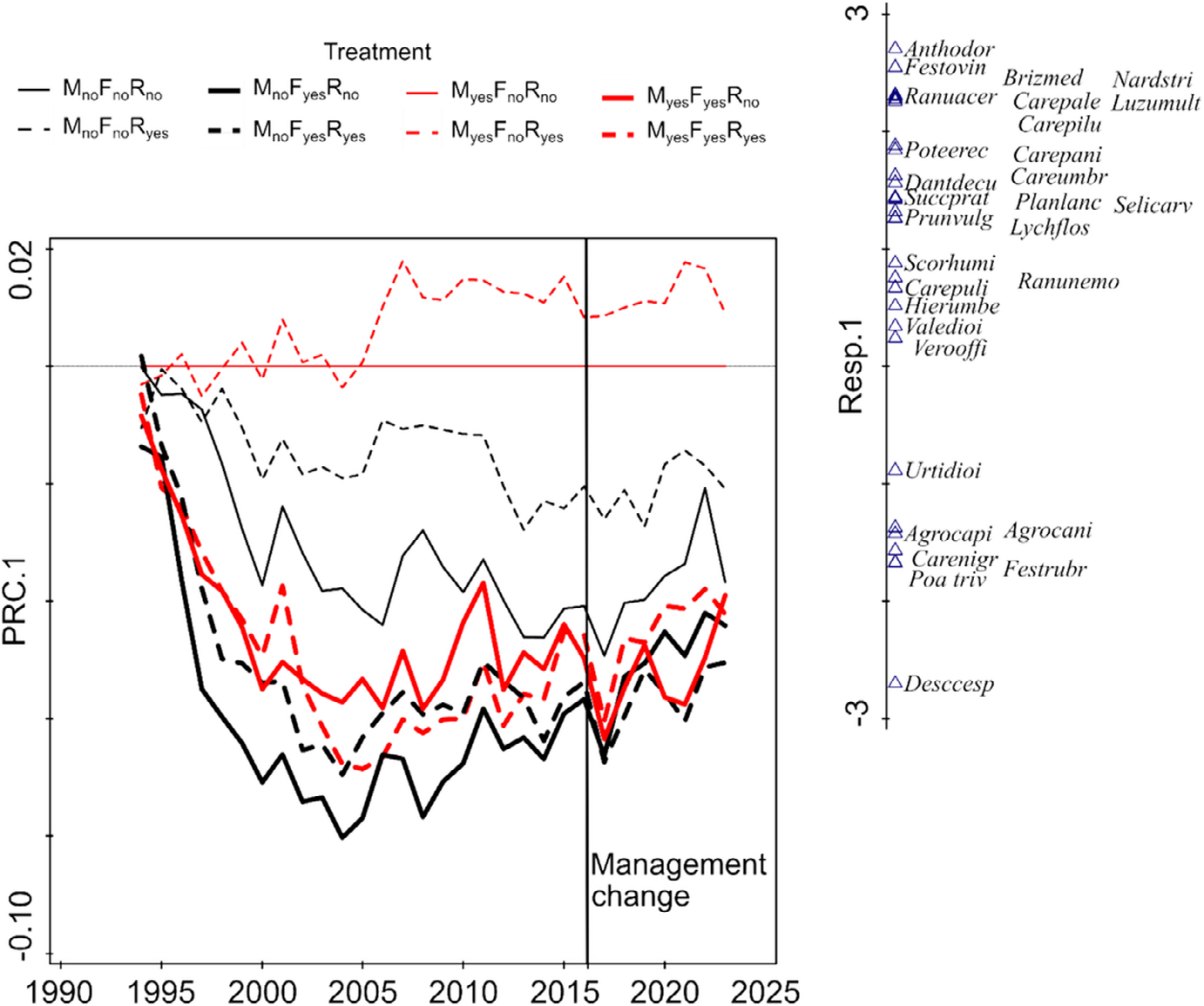
Principle response curves for individual treatment combinations. All the treatments are compared against the reference, which is mown unfertilized plot (shown by the abscissa). The vertical line shows the time when fertilization was abandoned and mowing resumption. The vertical axis on the right side shows scores of the species, meaning their preferences for individual treatment combinations. The heavy lines mean fertilization (light = no fertilization), the red lines mean mowing (black = unmown) and the broken lines mean *Molinia* removal. Full species names for the acronyms are in Table A1.

**FIGURE 3:**
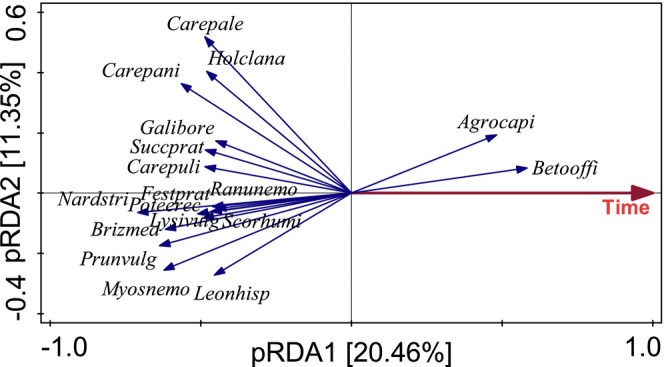
The results of partial RDA on the covariance matrix of the control plots. The temporal trend is highly significant (*p* = 0.0002 with 4999 permutations). Full species names for the acronyms are in Table A1. Values in parentheses are percentages of partial variability explained by individual axes. Only species with at least 20% fit on the first axis (i.e., species with the strongest temporal trend) are shown.

The relevés of all the plots at the baseline (i.e., 1994) were very similar to each other but began to differ immediately after introduction of the treatments (Figure 1). Fertilization was the main driver of the changes corresponding with the first axis of the CCA. For a limited time, mowing, and to a lesser extent dominant removal, partially mitigated the effect of fertilization. The effect of dominant removal was rather small, being most pronounced in unmown unfertilized plots where, without removal, *Molinia* attained high cover. The species which were harmed most by either fertilization or abandonment of mowing were low‐statured, non‐competitive species like *Anthoxanthum odoratum, Nardus stricta, Carex pallescens* (and several other sedges), *Briza media*, or *Potentilla erecta* (species scores in Figure 1 and Figure 2). After stopping fertilization and re‐introducing mowing, the fertilized and unmown plots started to slowly converge with the originally mown, unfertilized plots. The return was faster in the case of unmown unfertilized plots than in previously fertilized plots (both mown and unmown). The second axis explains less variability (Figure 1) and reflects a common temporal trend. This upward shift in the ordination diagram is also pronounced in the mown unfertilized plots (both with and without removal), i.e. in the plots that had the same treatment throughout the experiment. This is well illustrated by the highly significant effect of time in RDA of species composition for the three plots kept with traditional management (Figure 3).

**FIGURE A4:**
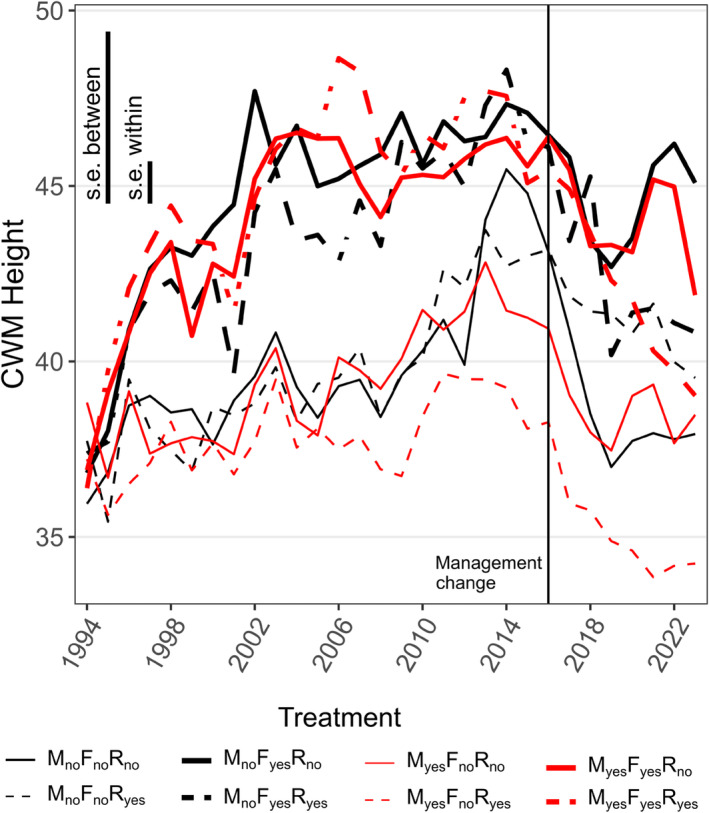
The temporal changes in CWM for height (all the species including *Molinia* are used). Results of corresponding Repeated measures ANOVA are in Table S4. The heavy lines mean fertilization (light = no fertilization), the red lines mean mowing (black = unmown) and the broken lines mean *Molinia* removal. The vertical lines mean standard errors for between and within plot comparisons, derived from the Repeated Measures ANOVA model (Table S4). The re‐installment of original traditional management in 2016 is shown by thin vertical line.

The principal response curves (Figure 2) show the differentiation according to the treatments in individual years. The overwhelming effect of fertilization (meaning the low resistance to fertilization) was partially mitigated by mowing and dominant removal; abandonment and removal effects were generally smaller but more pronounced in the unfertilized plots. The difference between the removal and non‐removal plots was clearly dependent on the cover of *Molinia* in non‐removal plots, i.e. highest in the unfertilized unmown plots (Figure A4). The differentiation increased during the first eight years of the experiment and then the differences stabilized. The majority of species were negatively affected by fertilization and absence of mowing. Only a few species were supported, notably *Urtica dioica* (originally absent from the locality) and *Scirpus sylvaticus* which was originally present as scattered individuals, but in fertilized unmown plots achieved high dominance. After the re‐establishment of traditional management, the return to the reference state was faster in the non‐fertilized plots; in all combinations including fertilized plots, return is detectable but slow and even after 9 years, the plots are far from the reference; the resilience after cessation of fertilization is thus very low.

**FIGURE 4:**
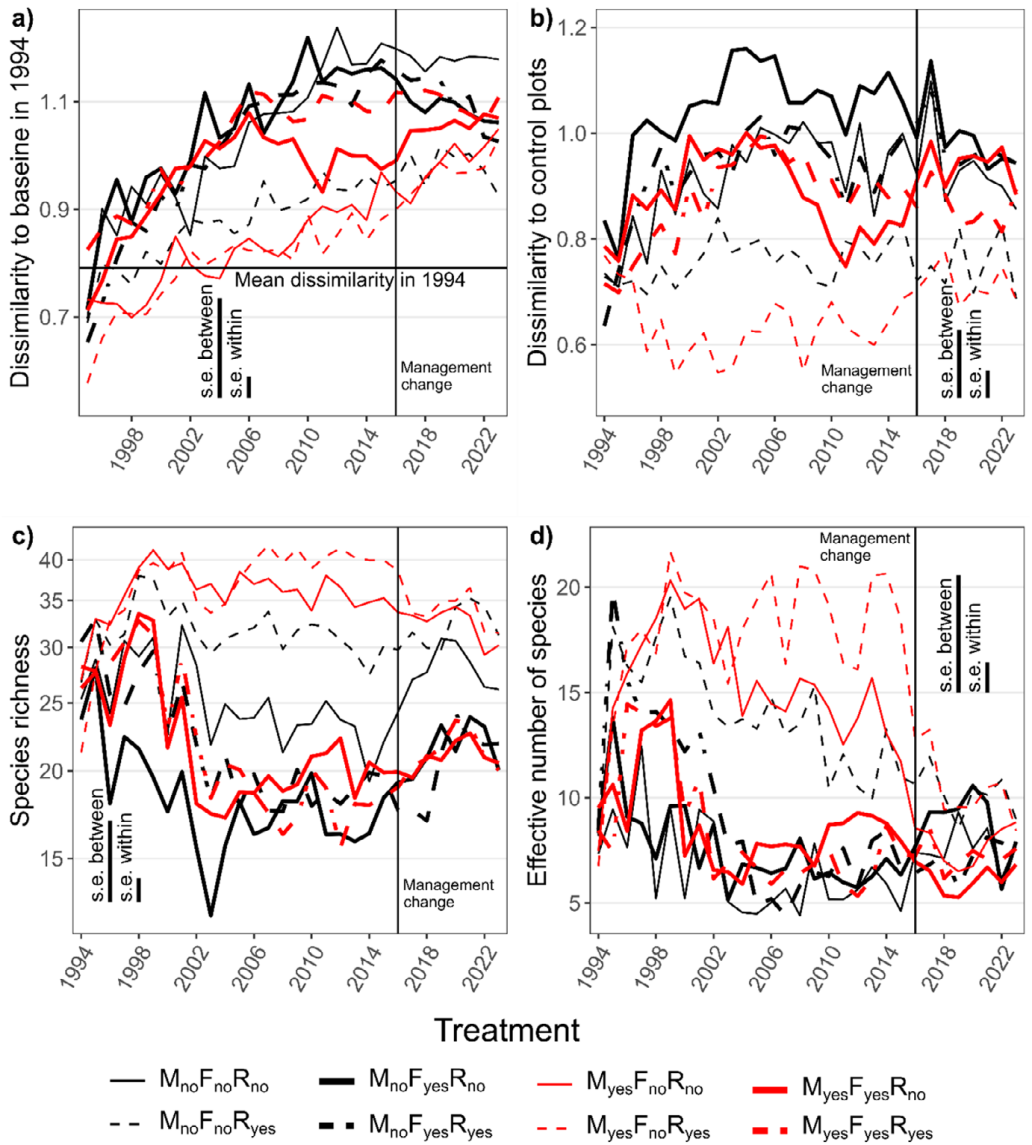
The temporal changes in (a) the chord dissimilarity to the baseline state in 1994 of each quadrat, (b) the chord dissimilarity to the control plots within each year, (c) the species richness, and (d) effective number of species (ENS). Results of corresponding repeated measures ANOVA are in Table A3. The heavy lines mean fertilization (light = no fertilization), the red lines mean mowing (black = unmown) and the broken lines mean *Molinia* removal. The vertical lines mean standard errors for between and within plot comparisons, derived from the Repeated Measures ANOVA model. The re‐installment of original traditional management in 2016 is shown by thin vertical line.

The general increase of the dissimilarity to the baseline state (Figure 4a) corresponds to the directional trend—the trend in the mown unfertilized plots was the slowest, but consistent. The fertilized plots showed the fastest increase, but these plots were the only ones where the dissimilarity decreased after re‐establishment of traditional management. The repeated measures ANOVA (Table A3) shows the strong effect of time (dissimilarity in average increases); of the experimental treatments, fertilization was the strongest factor. The effect of removal was weak, but it modified (mitigated) the effect of mowing abandonment. The unmown plots with removal showed very fast recovery and, at the end of the experiment, had the lowest dissimilarity to the baseline. The dissimilarity to control in a given year (Figure 4b) shows an immediate increase, particularly fast in all the fertilized plots, but the most pronounced in fertilized unmown plots. At the end of the first decade, the differentiation ceases and in some treatment combinations, it even decreases. The recovery after re‐introduction of traditional management is most visible in fertilized plots, but in all cases, the differences remain large even after 8 years of traditional management.

The species richness in mismanaged plots decreased (Figure 4c), with the fastest decline in the fertilized, unmown, no‐removal plots. During the first five years, it seemed that mowing could prevent a decrease in species richness but, shortly after the 10th year, all the fertilized plots fell to similarly low richness. Again, the dominant removal partially mitigated the negative effect of absence of mowing in unfertilized plots. After the resumption of mowing and end of fertilization, species richness started to increase. In the unfertilized, unmown plots the richness returned quite quickly. The return in the fertilized plots was much slower, and far from complete. The comparison is particularly striking when we compare the plots that were (in contrast to traditional management) only unmown and only fertilized.

The dynamics of ENS (Figure 4d) shows clear convergence of all the treatment combinations after the return of the traditional management; however, it is not caused by increase in mismanaged plots after the end of the mismanagement, but by a decrease in the controls, part of a generally decreasing trend independent of treatments. In all the plots including those with removal, some of the already present species achieved high cover (in non‐fertilized plots often *Danthonia decumbens* ), decreasing evenness and thus also ENS. The effect of removal on ENS was more pronounced than on species richness, mainly due to *Molinia's* dominance. The changes in CWM for vegetative height (Figure A4, Table A4) showed a fast increase with fertilization, and decreased after resuming the traditional management.

**FIGURE A5:**
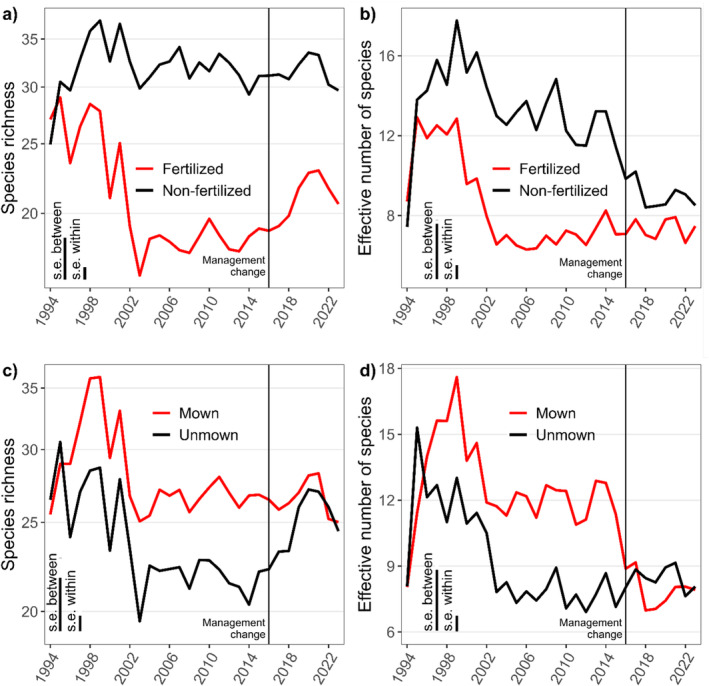
Marginal effect of Fertilization and Mowing on the species richness and Effective Number of Species (ENS) during the experiment time.

The contrasts in resistance and resilience of species richness and ENS to fertilization and mowing abandonment are also clearly visible from the temporal dynamics of marginal effects (effect of each factor averaged over all the other treatments). For both richness and ENS, fertilization had an effect much sooner and more pronounced than that of mowing, but the differences stabilized after ~10 years for species richness (Figure A5). After a return to traditional management, the recovery of species richness after re‐introduction of mowing was fast (i.e., high resilience), the effect of fertilization, however, lasted.

**FIGURE A6:**
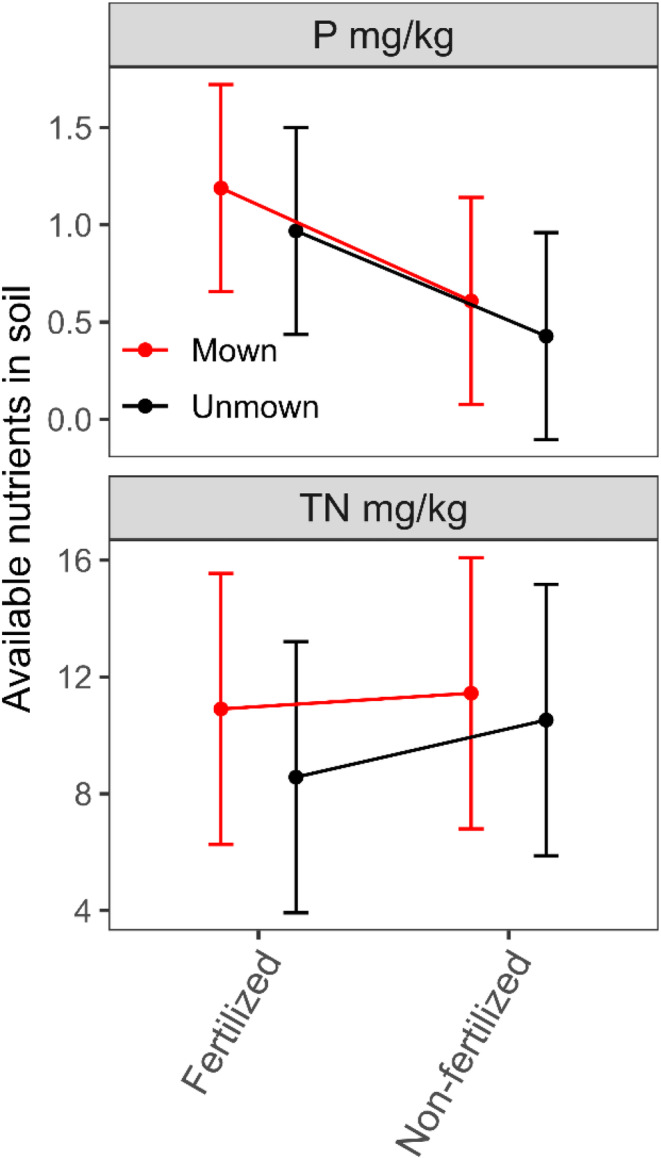
Available Phosphorus and Nitrogen in the soil in 2022, that is, 6 years after resumption of the traditional management (mowing and no fertilization).

One of the reasons for low resilience after fertilization might be the environmental legacy effects. A soil analyses done in 2022 (i.e., 6 years after end of the perturbation period) showed no differences between mowing and removal plots, but the previously fertilized plots still had significantly higher levels of available Phosphorus (Figure A6), but no differences in Nitrogen.

## 4 Discussion

This long‐term experiment in a species rich, traditionally managed meadow demonstrates that both introduction of fertilization and abandonment of mowing have detrimental effects on species composition, also causing strong diversity decrease. The resistance of species composition and diversity to mowing abandonment is stronger than to fertilization. The resumption of traditional management led to the return of (some) species and was faster in previously unmown plots without fertilization. The low resilience of fertilized plots was probably caused by the legacy effects of fertilization. The removal of the dominant *Molinia* was able to partially mitigate the adverse effects of mowing abandonment, but only in unfertilized plots. There was a clear common trend in species composition and decrease of diversity in all the treatment combinations, likely caused by environmental changes driven by global warming.

Resistance and resilience characteristics are dependent on the perturbation type. It has already been shown that the contrast in resistance of two successional stages depended on perturbation type (Koblihová‐Baumová et al. 1990; Lepš 1990) and also on perturbation intensity, not studied here. In our case, resistance irrespective of state variables considered and resilience of species richness were higher for abandonment than for fertilization; nevertheless, both fertilization and abandonment led to the loss of low‐statured species, apparently as a result of increased asymmetry in competition connected with the replacement of belowground with aboveground competition (Lepš 1999). Nearly no new species appeared in fertilized and abandoned plots. The only exception was *Urtica dioica*, which was previously absent from the plots and became dominant in some fertilized, unmown plots. *Urtica* disappeared rather quickly with resumption of mowing and cessation of fertilization (similarly fast retreat was demonstrated by Prach 2007).

There was a concordant increase in species richness in all the treatments from the first to the second year. Only in the next year did the richness start to change in response to the treatments (Figure 4c). Interestingly, there was no common trend from the first to the second year in species composition, while differentiation according to the treatments began in the first year (Figure 1). Apparently, the first effect of the treatments was changing proportions of individual species, and extirpation started only from the second year on. Probably, we also learned to avoid overlooking inconspicuous species with extremely low cover (similarly as Lisner and Lepš 2020) during the first sampling—these species had a negligible effect on ordinations, but affected the total number of species. The difference between dynamics of species richness and ENS suggests that changes in proportions of species are faster and are followed by individual species local extinction later on. The pronouncedly higher ENS in removal plots in comparison with corresponding management combinations with *Molinia* present showed that none of the remaining species were able to replace *Molinia* as a dominant during the whole mismanagement period, but some of the species became new dominants due to the decrease of groundwater level (next paragraph).

Our analyses revealed a clear shift in species composition, also in plots with constant management. As a consequence, the resistance and resilience evaluation depend on whether the reference was the traditional management in given year (as in PRC, Figure 3 and chord distance to control, Figure 4b), or the baseline data for given quadrat (Figure 4a). Global warming (see the continuous increase in temperature in Figure A1) is the suspected factor causing this trend (Liu et al. 2018). Based on our observations, we expect that the reason is not temperature itself, but a decrease in the ground‐water level caused by global warming. The omnipresent trend in species composition is confirmed by analysis of repeated observations of permanent plots of Central European non‐forest vegetation (Klinkovská et al. 2025).

It seems that the main driver of species loss is competitive exclusion; the best predictor of a species decline after fertilization and mowing abandonment was its small vegetative height (Lepš 1999, 2014). Accordingly, the CWM (calculated using fixed values) for height increased in fertilized (and also unmown) plots and decreased after resumption of traditional management (Figure A4). Fertilization releases plants from the nutrient competition underground, but leads to increased competition for light (Wilson and Tilman 1993), which is much more asymmetric and thus leads to exclusion of low species (Lepš 1999). Cessation of mowing had a similar effect. Regular mowing (at least partially) reverses this competition asymmetry, because mowing harms tall, erosulate growth form plants more than low plants with rosettes (Klimešová et al. 2008). After resumption of mowing, particularly in unfertilized plots, the competition asymmetry was immediately decreased and species richness started to increase. Correspondingly, Yamada et al. (2024) also found very fast recovery after restoration of mowing in a dry grassland (however, their abandonment period was much shorter than our one) or Prach (2007) in floodplain meadows. In contrast, the increase of species richness after cessation of fertilization is much slower. Probably the most important reason for this is the environmental legacy of fertilization (Figure A6), as some of the dominants are still able to take advantage of increased phosphorus in the soil. Some dominants in fertilized plots, particularly grasses (also in the fertilized mown plots) were able to keep their high abundance; very probably, for strong competitors the preemptive effect in the competition for space results in founder controlled communities (Yodzis 1978), where the penetration of subordinate species is very slow. It has been shown that the environmental legacy of fertilization hinders the return to the “reference” species composition for decades (Isbell et al. 2013; Seabloom et al. 2020; Spiegelberger et al. 2006). The return of species composition in non‐fertilized abandoned plots towards the traditionally managed control is faster than in fertilized plots according to PRC (Figure 2); however, the situation is less clear when evaluated by the chord distance to baseline (see the discussion on methodological problems below).

**FIGURE A7:**
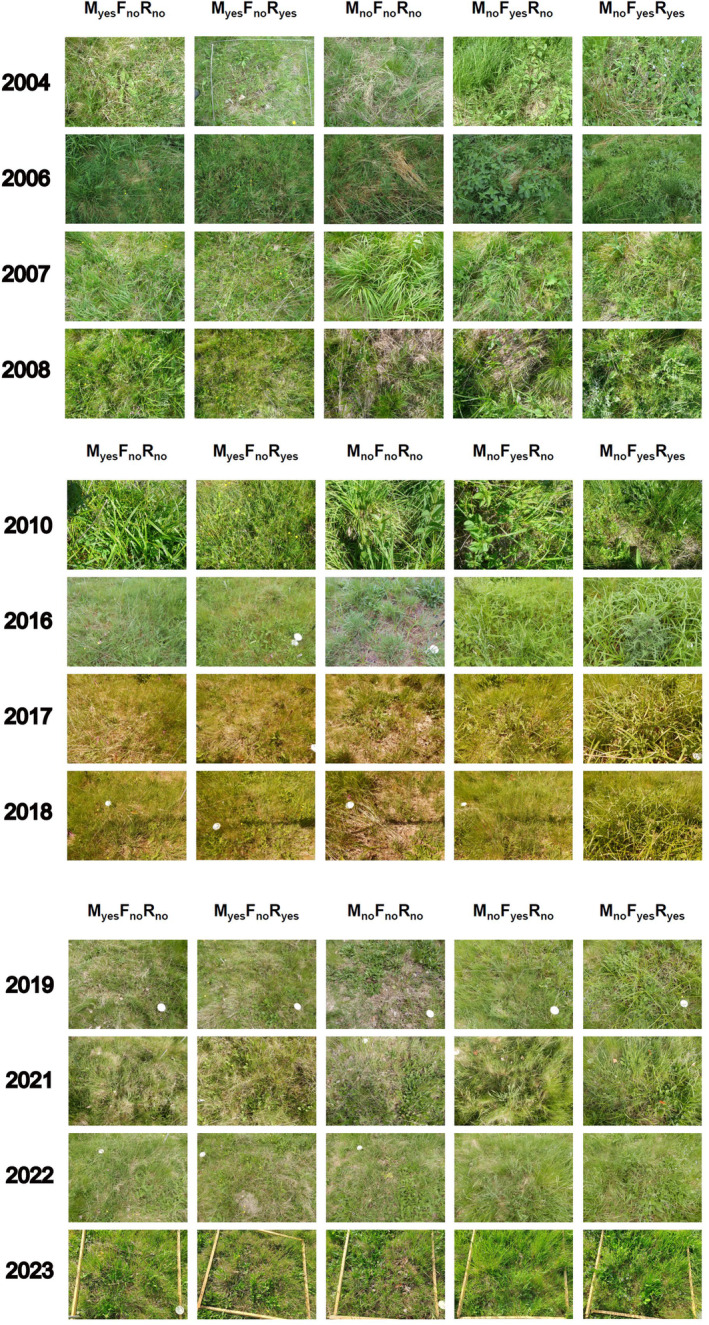
The state of five selected plots (columns) in selected years (rows). Five plots representing five treatment combinations are selected (M, F, and R indicate Mowing, Fertilization, and Removal respectively). M_yes_F_no_R_no_ (plot 11) corresponds to traditional management, M_yes_F_no_R_yes_ (plot 21) is the most species rich combination, M_no_F_no_R_no_ (plot 2) represents total abandonment. The two rightmost columns M_no_F_yes_R_no_ (plot 13) and M_no_F_yes_R_yes_ (plot 19) are eutrophicated abandoned plots, (plot 13) without dominant removal and (plot 19) with. The series starts in 2004, that is, 10 years after the introduction of the mismanagement perturbation period. Note how *Molinia caerulea* attains high dominance in the abandoned plot (3rd column from the left), which is followed by a strong decline after re‐introduction of mowing in 2016. In the unmown fertilized plots (the two rightmost columns), note the appearance of strong dominants, which attain high dominance towards the end of the mismanagement period, that is, in 2016, and retreat after re‐introduction of traditional management. *Urtica dioica* nearly disappeared in only 2 years, whereas *Scirpus sylvaticus* decreased slower, and even in 2023 remains as a subordinate species with low cover, similar to the original community.

According to some theories, community functioning (and thus stability characteristics) depends on the species pool perhaps more than on the actual species composition (Hagan, Vanschoenwinkel, and Gamfeldt 2021), which stresses the role of the newly invading species. *Urtica dioica* invaded the fertilized unmown plots and attained high dominance. Its spread was probably reinforced by its very efficient clonal behavior (Hara and Šrutek 1995). However, after re‐establishment of cutting and without fertilization, this species very quickly declined and, in most plots, completely disappeared (Figure A7). The species is known to be very sensitive to cutting, but it is able to survive cutting in soil with a high concentration of Nitrogen, whereas a high concentration of Phosphorus generally does not help (Müllerová et al. 2014). Another species able to dominate the unmown, fertilized plots was *Scirpus sylvaticus* , another species with efficient clonal spread. Through its very dense canopy and recalcitrant litter (personal observation), it is able to suppress most of the subordinate species. After re‐introduction of mowing, it became a scattered species quite quickly, but did not disappear completely (Figure A7). The dynamics of these two species, which were able to drastically suppress diversity, demonstrates the importance of availability of potential dominants in the community or in the species pool. The resistance and resilience of a community are very much affected by the traits of its constituent species (Lepš, Osbornová‐Kosinová, and Rejmánek 1982), particularly by the often idiosyncratic trait combinations of dominants (Lisner et al. 2023, 2024), potential dominants already present in the species pool (Hagan, Vanschoenwinkel, and Gamfeldt 2021), and/or the ability of dominants to reach the site. We can only speculate on how much the invasion of *Urtica* was facilitated by regular visits of researchers, serving as seed dispersers. The dominant removal does not substantially change neither the species composition nor its resistance and resilience characteristics. This is surprising, because *Molinia* is able to compensate for all other species lost from the community (as shown in a complementary experiment on the same site, Lisner et al. 2023).

Isbell et al. (2015) suggested formulae, which quantify resistance and resilience on the basis of differences between individual states before, during, and after perturbation. In comparison with their approach, we face two main problems—the multivariate nature of species composition and the temporal trend also affecting the reference. We solved the multivariate nature in two ways—using the framework of (constrained) ordinations and using the compositional dissimilarity index instead of the absolute value of plain difference in Isbell et al. (2015), and calculated the distance to two reference states. By following the directional trend, we have demonstrated the difference in the use of the reference state before the perturbation period (as shown in Figure 4a), versus focusing on the state of a selected reference management (i.e., traditional management) at each time of observation (as implicitly represented in the PRC and explicitly depicted in Figure 4b).

The recovery after resumption of traditional management in unmown plots is, according to PRC (i.e., the return to the species composition towards the reference plots in corresponding year), well documented and relatively fast. However, differences to starting species composition in reference plots mostly increase also after the re‐establishment of traditional management—the return is overridden by the general trend of all the plots.

In the case of perturbation of the press type (Polazzo and Rico 2021), the resistance changes according to the time elapsed from the start of the perturbation period—typically, the resistance of mown plots to fertilization seems to be high for up to 3 years, but then decreases tremendously. The resistance includes the speed of the change and its extent. This is demonstrated for species richness, where the rate of decrease is much higher for fertilized than for abandoned plots, however, after 10 years, both fell to roughly same level. It's a similar case for the return after the perturbation period (of any type) ends. Resistance and resilience characteristics are related to the time scale of corresponding response (Lepš 1990). The return might be modeled as an asymptotic return, however, the reality is usually more complicated than these elegant models; the return interferes with the common trend, but also with the year‐to‐year variability. For the species richness, the re‐introduction of traditional management leads to an increase in species richness (and thus convergence with the control), but for species composition, the year‐to‐year variability (Figures 2 and 4b) shows that the return to the reference is far from monotonous (and for species composition, the dumped oscillations suggested by Isbell et al. 2015 also do not work well). Nevertheless, even on the basis of these models, Arnoldi et al. (2018) differentiated instantaneous and average rates of return.

## 5 Conclusion

From a methodological point of view, the resistance and resilience assessment always reflects: (1) the type of perturbation, and potential legacies (2) the “state variables” measured, and (3) the temporal scales of responses. In a long‐term data series, temporal trends are likely (Klinkovská et al. 2025), and thus, the assessment will also reflect our definition of reference state. The stability characteristics are affected not only by constituent species, but also by potentially invading species from the local species pool.

From a conservation point of view, it is much easier and faster to destroy the biodiversity of species rich communities than to restore it, and in some cases, full restoration might not be feasible. Consequently, the protection of species rich communities should be the conservation priority. In this case, fertilization legacy effects will be the main obstacle to the return of biodiversity, should we try to restore it.

From our experience, most long‐term projects start as short‐term experiments, extended only when results become interesting. Our project is a typical example. When JL established the experiment in 1994, he did not anticipate the strong effects of global warming or the groundwater table's decline. While it would be ideal to plan long‐term experiments from the start, it's practical to consider the possibility of extension when designing short‐term studies.

## Author Contributions

**Jan Lepš:** conceptualization (lead), data curation (equal), formal analysis (equal), funding acquisition (lead), investigation (lead), methodology (lead), project administration (equal), resources (equal), visualization (supporting), writing – original draft (lead), writing – review and editing (equal). **Aleš Lisner:** conceptualization (supporting), data curation (equal), formal analysis (equal), investigation (supporting), methodology (supporting), project administration (equal), visualization (lead), writing – original draft (supporting), writing – review and editing (equal).

## Conflicts of Interest

The authors do not have any conflicts of interest to report.

## Supporting information


Data S1.

## Data Availability

Data are stored in the Dryad Digital Repository https://doi.org/10.5061/dryad.1g1jwsv78, and also provided as a “Supporting Information” file.

## Appendix A

**TABLE A1:** Acronyms and corresponding species names used in ordination diagrams (Figures 1, 2, 3). The nomenclature follows the database of the Czech flora PLADIAS (https://pladias.cz/), accessed on May 14, 2024.

Achiptar	*Achillea ptarmica*
Agrocani	*Agrostis canina*
Agrocapi	*Agrostis capilaris*
Angesylv	*Angelica sylvestris*
Anthodor	*Anthoxanthum odoratum*
Betooffi	*Betonica officinalis*
Brizmed	*Briza media*
Carehart	*Carex hartmanii*
Carenigr	*Carex nigra*
Carepale	*Carex palescens*
Carepani	*Carex panicea*
Carepilu	*Carex pilulifera*
Carepuli	*Carex pulicaris*
Careumbr	*Carex umbrosa*
Cirspalu	*Cirsium palustre*
Dantdecu	*Danthonia decumbens*
Desccesp	*Deschampsia cespitosa*
Festovin	*Festuca ovina*
Festprat	*Festuca pratensis*
Festrubr	*Festuca rubra*
Galibore	*Galium boreale*
Hierumbe	*Hieracium umbellatum*
Junceffu	*Juncus effusus*
Leonhisp	*Leontodon hispidus*
Luzumult	*Luzula multiflora*
Lychflos	*Lychnis flos‐cuculi*
Lysivulg	*Lusimachia vulgaris*
Myosnemo	*Myosotis nemorosa*
Nardstri	*Nardus stricta*
Pimpmajo	*Pimpinella major*
Planlanc	*Plantago lanceolata*
Poatriv	*Poa trivialis*
Poteerec	*Potentilla erecta*
Prunvulg	*Prunella vulgaris*
Ranuacri	*Ranunculus acris*
Ranunemo	*Ranunculus nemorosus*
Rumeacet	*Rumex acetosa*
Scirsylv	*Scirpus sylvaticus*
Scorhumi	*Scorzonera humilis*
Selicarv	*Selinum carvifolia*
Succprat	*Succisa pratensis*
Urtidioi	*Urtica dioica*
Valedioi	*Valeriana dioica*
Verooffi	*Veronica officinalis*

**TABLE A2:** Results of the Repeated measures ANOVA for the Cover of *Molinia*–only the non‐removal plots were analyzed.

	NumDF, DenDF	*F*	*p*
Year	29, 232	**6.083**	**< 0.001**
Mowing	1, 8	3.661	0.092
Fertilization	1, 8	**12.443**	**0.008**
Year × Mowing	29, 232	**5.327**	**< 0.001**
Year × Fertilization	29, 232	**2.054**	**0.002**
Mowing × Fertilization	1, 8	1.376	0.275
Year × Mowing × Fertilization	29, 232	0.365	0.999

*Note:* Significant relationships (*p* < 0.05) are highlighted in bold.

Abbreviation: NumDF, DenDF–numerator and denominator degrees of freedom.

**TABLE A3:** Results of the Repeated measures ANOVA for Dissimilarity to baseline, Dissimilarity to control plots in given year, Species richness and Effective number of species.

	Dissimilarity to baseline in 1994			Dissimilarity to control plots			NumDF, DenDF	Species richness		Effective number of species	
	NumDF, DenDF	*F*	*p*	NumDF, DenDF	*F*	*p*		*F*	*p*	*F*	*p*
Year	28, 448	**49.517**	**< 0.001**	29, 406	**5.888**	**< 0.001**	29, 464	**11.877**	**< 0.001**	**17.139**	**< 0.001**
Mowing	1, 16	**11.389**	**0.004**	1, 14	**29.265**	**< 0.001**	1, 16	**17.810**	**< 0.001**	**10.050**	**0.006**
Fertilization	1, 16	**20.593**	**< 0.001**	1, 14	**71.306**	**< 0.001**	1, 16	**151.379**	**< 0.001**	**31.815**	**< 0.001**
Removal	1, 16	2.666	0.122	1, 14	**21.575**	**< 0.001**	1, 16	**6.702**	**0.020**	**11.183**	**0.004**
Year × Mowing	28, 448	**2.981**	**< 0.001**	29, 406	**1.652**	**0.020**	29, 464	**2.725**	**< 0.001**	**6.004**	**< 0.001**
Year × Fertilization	28, 448	**3.282**	**< 0.001**	29, 406	**2.327**	**< 0.001**	29, 464	**9.358**	**< 0.001**	**5.595**	**< 0.001**
Mowing × Fertilization	1, 16	4.042	0.062	1, 14	0.533	0.477	1, 16	**4.712**	**0.045**	**11.636**	**0.004**
Year × Removal	28, 448	0.617	0.939	29, 406	0.735	0.842	29, 464	1.134	0.290	**1.911**	**0.003**
Mowing × Removal	1, 16	**5.052**	**0.039**	1, 14	2.168	0.163	1, 16	**4.929**	**0.041**	2.336	0.146
Fertilization × Removal	1, 16	**4.933**	**0.041**	1, 14	2.314	0.151	1, 16	0.937	0.347	**7.401**	**0.015**
Year × Mowing × Fertilization	28, 448	0.633	0.929	29, 406	1.166	0.256	29, 464	**2.116**	**< 0.001**	**2.441**	**< 0.001**
Year × Mowing × Removal	28, 448	0.779	0.785	29, 406	1.364	0.102	29, 464	1.160	0.262	**2.831**	**< 0.001**
Year × Fertilization × Removal	28, 448	**1.641**	**0.022**	29, 406	1.192	0.230	29, 464	**2.262**	**< 0.001**	**2.015**	**0.002**
Mowing × Fertilization × Removal	1, 16	0.345	0.565	NA	NA	NA	1, 16	0.151	0.703	0.703	0.414
Year × Mowing × Fertilization × Removal	28, 448	1.145	0.280	NA	NA	NA	29, 464	0.510	0.985	0.800	0.764

*Note:* Significant relationships (*p* < 0.05) are highlighted in bold.

Abbreviation: NumDF, DenDF–numerator and denominator degrees of freedom.

**TABLE A4:** Results of the Repeated measures ANOVA for CWM for height.

	NumDF, DenDF	*F*	*p*
Year	29, 464	**20.357**	**< 0.001**
Mowing	1, 16	1.269	0.277
Fertilization	1, 16	**63.229**	**< 0.001**
Removal	1, 16	1.420	0.251
Year × Mowing	29, 464	**1.709**	**0.013**
Year × Fertilization	29, 464	**3.445**	**< 0.001**
Mowing × Fertilization	1, 16	1.913	0.186
Year × Removal	29, 464	1.087	0.348
Mowing × Removal	1, 16	0.175	0.681
Fertilization × Removal	1, 16	0.005	0.945
Year × Mowing × Fertilization	29, 464	0.730	0.848
Year × Mowing × Removal	29, 464	1.156	0.266
Year × Fertilization × Removal	29, 464	1.228	0.195
Mowing × Fertilization × Removal	1, 16	2.652	0.123
Year × Mowing × Fertilization × Removal	29, 464	0.608	0.948

*Note:* Significant relationships (*p* < 0.05) are highlighted in bold.

Abbreviation: NumDF, DenDF–numerator and denominator degrees of freedom.
